# The Ectomycorrhizospheric Habitat of Norway Spruce and *Tricholoma vaccinum*: Promotion of Plant Growth and Fitness by a Rich Microorganismic Community

**DOI:** 10.3389/fmicb.2019.00307

**Published:** 2019-02-20

**Authors:** Katharina Wagner, Katrin Krause, Ramses Gallegos-Monterrosa, Dominik Sammer, Ákos T. Kovács, Erika Kothe

**Affiliations:** ^1^Microbial Communication, Institute of Microbiology, Friedrich Schiller University Jena, Jena, Germany; ^2^Terrestrial Biofilms Group, Institute of Microbiology, Friedrich Schiller University Jena, Jena, Germany

**Keywords:** ectomycorrhiza, community, microcosm, indole-3-acetic acid, *Tricholoma*, plant growth promoting bacteria

## Abstract

The contribution of the mycorrhizospheric microbes in a stand of ectomycorrhizal Norway spruce (*Picea abies*) featuring mycorrhiza with the basidiomycete *Tricholoma vaccinum* was addressed by microbiome analysis and *in vitro* reconstruction of microbial as well as plant-microbe interactions. The protective role of the mycorrhizal fungus with respect to pathogen attack could be validated against *Botrytis cinerea* and *Heterobasidion annosum* in co-cultures revealing reduced pathogen growth, higher survival rate of the spruce trees and reduced symptoms on needles upon symbiosis with *T. vaccinum.* The community structure was shown to yield a high diversity in ECM forming basidiomycetes of *Thelephorales* and *Agaricales* associated with a rich bacterial diversity dominated by *Rhizobiales* with the most abundant *Nitrobacter winogradski* (3.9%). Isolated bacteria were then used to address plant growth promoting abilities, which included production of the phytohormone indole-3-acetic acid (performed by 74% of the bacterial isolates), siderophores (22%), and phosphate mobilization (23%). Among the isolates, mycorrhiza helper bacteria (MHB) were identified, with *Bacillus cereus* MRZ-1 inducing hyperbranching in *T. vaccinum*, supporting tree germination, shoot elongation, and root formation as well as higher mycorrhization rates. Thus, a huge pool of potential MHB and fungal community with widely distributed auxin-production potential extended the ability of *T. vaccinum* to form ectomycorrhiza. The forest community profited from the mycorrhizal fungus *T. vaccinum*, with spruce survival enhanced by 33% in microcosms using soil from the native habitat. A higher fungal abundance and diversity in cases where the tree had died during the experiment, showing that decomposition of plant litter from a dead tree supported a different community. *T. vaccinum* thus actively structured the community of microorganisms in its habitat.

## Introduction

Forest ecosystems are stabilized by the mutual symbiosis between tree roots and mainly basidiomycete fungi forming the ectomycorrhizal symbiosis with nutrient and water supply to the roots in exchange for photosynthesis products from the tree. In the specific mutualistic symbiosis between mainly basidiomycete fungi and trees, the ectomycorrhiza (ECM), the fungal partner forms a mantle around the short roots of its host, which has been suggested to protect the tree from pathogen attack (Smith and Read, 1987). In addition, hyphae grow between the rhizosphere cells forming the Hartig’ net which is the interface for nutrient and signal exchange (for review, see Raudaskoski and Kothe, 2015). Since mycorrhiza is known to increase tree fitness, the late stage basidiomycete *Tricholoma vaccinum* was chosen for investigation. It forms host specific spruce ectomycorrhiza (Asiimwe et al., 2012). *T. vaccinum* produces the auxin phytohormone indole-3-acetic acid (IAA), which promotes higher branching and increases hyphal lengths of the mycelium, combined with an increased Hartig’ net formation during symbiosis (Krause et al., 2015).

However, additional functions may be supplied by the rich microbiota present in the surrounding soil. To address interactions in the ectomycorrhizosphere, the microbiome of this habitat needs to be addressed with specific regard to the ectomycorrhiza. Here, *Tricholoma vaccinum* and Norway spruce (*Picea abies*) were investigated and the community assessed in a natural stand of spruce/*T. vaccinum*.

Spruce is the most common tree in Europe, making up over 30% of German and Switzerland forests, and 10% of the total land area are covered by *P. abies* owing to forestry management (Klimo et al., 2000). Spruce pathogens include *Botrytis cinerea* leading to blight especially in seedlings, and *Heterobasidion annosum* leading to root and butt rot. The gray mold *B. cinerea* is a virulent and common plant pathogen. It shows low host selectivity and mainly infects the needles of conifers (Williamson et al., 2007). Counter-acting induction of systemic acquired resistance involves up-regulation of salicylic acid, shown to be produced by, e.g., the fungus *T. vaccinum* in pure culture (Wagner, 2016). Therefore, an effect of mycorrhiza on protecting young seedlings in a systemic manner can be tested. On contrast, *H. annosum* is a typical forest root pathogen (Lundén et al., 2015). Two types, one more prevalent on pine (P type on *Pinus* species) and one on fir or spruce (S/F on species of *Abies/Picea*; see Asiegbu et al., 2005) have been assigned. The second type thus can be used to study root pathogenic fungi for a protective effect of mycorrhiza. The different strategies employed by these two pathogens lend themselves for investigation of beneficial interactions protecting the tree.

In addition, other saprophytic and parasitic fungi, as well as bacteria, archaea, protists, nematodes or viruses are present in forest soil (Bais et al., 2004). This microbial community structure and diversity is considered an important factor in responding to anthropogenic or other ecosystem disturbances (Baldrian et al., 2012). In coniferous forest soils, fungi are dominant in plant litter decomposition in the upper horizon (Buee et al., 2009), and competition within the diverse fungal community would affect fungal pathogens (Alabouvette, 1990). Bacteria more often are involved in the biogeochemical cycles in lower horizons (Bååth and Anderson, 2003). The habitat around the mycorrhized roots with its microbiota is defined as mycorrhizosphere (Johansson et al., 2004). The chemical communication in the mycorrhizosphere represents a complex process and includes diverse phytohormones, pheromones and various allelochemicals with different structures, e.g., steroids and proteins (Raudaskoski and Kothe, 2015, and citations therein).

Mycorrhiza helper bacteria (MHB) can enhance mycorrhiza establishment or increase mycorrhization strength on an individual plant (Deveau et al., 2012; Wu et al., 2012). MHBs can impact phosphate and iron nutrition (Ahmad et al., 2008), and they can mediate plant defense by signals supporting mycorrhiza formation (Wang et al., 1993). Other plant growth promoting bacteria are also present in the rhizosphere and can supply traits like nitrogen fixation, phosphate mobilization, antifungal or antibacterial properties, as well as cyanide, phytohormone, or siderophore synthesis (Saharan and Nehra, 2011). The phytohormone indole-3-acetic acid (IAA) is required for cell growth and differentiation in the plant and stimulates Hartig’ net development (Gea et al., 1994). Therefore, microorganisms that are able to produce IAA (or IAA inhibiting compounds) are able to modulate ectomycorrhization (Tsavkelova et al., 2006; Hause and Schaarschmidt, 2009).

Here, we aimed to characterize the natural mycorrhizosphere community of soil below *T. vaccinum* fruiting bodies found with their host *P. abies.* The influence of the community was investigated in microcosm experiments, and the response of the community to tree death from an ectomycorrhizosphere to a forest soil community was evaluated. Isolated community members were checked for their influence on either symbiotic partner *in vitro*, and plant growth promoting as well as specific mycorrhiza helper functions were verified. In co-cultivation, the protection of spruce against two known pathogens was shown for inoculation with *T. vaccinum*. With this combined approach, we could show the impact of *T. vaccinum* on the community structure, identify potential functions of the mycorrhizosphere community and link both to spruce health.

## Materials and Methods

### Soil Sampling and Characterization

Soil was sampled in the rhizosphere of two spruce trees in a forest dominated by spruce and pine trees near Jena, Germany at a marked point with the coordinates 50°55′10.8”N 11°31′30.4”E at different time points over 3 years to validate the general mycorrhizosphere community stability and response to seasonal changes. The samples were taken, where *T. vaccinum* fruiting bodies had been found and morphotyping had validated occurrence of *T. vaccinum* (Supplementary Figure S1). Sampling dates for sequential extraction were in October 2012, for the investigation of fungal community in March 2014 and for the investigation of bacterial community at six time points in October 2012, April 2013, October 2013, April 2014, October 2014, and April 2015. Soil was taken from the organic horizon (O) with litter and humus rich zones (L and H horizon) in 1–10 cm depth. The soil was transferred immediately to the laboratory at 4°C and sieved with a 3–4 mm mesh to eliminate bigger plant material.

The pH was measured as described by Minasny et al. (2011). For sequential extraction, soil was air-dried and sieved to <2 mm. Extraction was performed in triplicates for the mobile (F1) and specifically absorbed (F2) fraction for the biomobile and bioavailable element contents (Supplementary Table S1, Schütze et al., 2014). Sequential extraction and analysis of the carbon (C), sulfur (S), and nitrogen (N) contents in the soil samples were performed as described earlier (Schütze et al., 2013).

### DNA Extraction, Pyrosequencing, and Bioinformatics Community Evaluation

Total DNA of the microbial community was isolated using the MoBio Soil DNA Extraction Kit (MoBio Laboratories, Carlsbad, CA, United States) with four extractions of 0.3 g soil each to gain a representative result. DNA was stored at -20°C, and samples sent to GATC Biotech (Konstanz, Germany) for pyrosequencing after amplification of bacterial 16S rRNA (using primers 27F: AGA GTT TGA TCC TGG CTC AG and 534R: ATT ACC GCG GCT GCT GG) or fungal ITS1 (primers ITS1F: CTT GGT CAT TTA GAG GAA GTA A and ITS2: GCT GCG TTC TTC ATC GAT GC; Gardes and Bruns, 1993). The reason for using ITS2 was to show broader coverage of fungal lineages as compared to using the ITS4 primer (Ihrmark et al., 2012). FLX titanium and paired-end Illumina sequencing was used. Non-chimeric unique clusters (see Supplementary Table S2 for read statistics and OTU assignment) were used for BLASTn analysis with an *E*-value cutoff of 1e-06 by non-redundant ITS references from UNITE database (updated on February, 2014) for fungi and 16S rDNA sequences from Ribosomal Database Project (RDP release 11, updated on March 2014; Cole et al., 2009). Sequences were deposited at GenBank MG255224-MG255268 (fungal ITS) and at GenBank MG282098-MG282149 (bacterial16S rRNA). Sequences for all representative species were retrieved from the mentioned databases and aligned using MAFFT online (version 7)^1^. The alignment was corrected manually, a neighbor-joining tree constructed and data bootstrapped using MrBayes (Bayesian Inference of Phylogeny, version 3.2)^2^ with 6 Mio generations for bacteria and 1 Mio generations for fungi. Rarefaction was controlled (see Supplementary Figure S2).

### Isolation of Bacteria and Identification

A dilution series in 0.9% NaCl was plated on Standard I medium (StdI; Merck, Darmstadt, Germany). After incubation at 28°C for 2 days, colony forming units (CFU/g soil) were determined. Morphologically different isolates were selected as operational taxonomic units (OTUs) for further analyses. From the total OTUs, 94 were randomly selected for further identification. Fast DNA isolation was achieved by boiling a loop of biomass in 50 μl distillated water for 5 min. DNA was isolated using CTAB preparation (Ausubel et al., 1992). 16S rDNA sequence was amplified with the primers A1 (GAG TTT GAT CAT GGC TCA) and B6 (TTG CGG GAC TTA ACC CAA CAT) using PCR (program: 95°C for 3 min, 35 cycles of 95°C 30 sec, annealing at 52°C for 45 s, elongation at 72°C for 1 min 30 s, and final extension at 72°C for 10 min). Fragments were eluted from agarose gel and directly used for sequencing at GATC (Konstanz, Germany) or cloned into pGEM-TEasyTM vector (Promega, Madison, United States) followed by transformation into competent *Escherichia coli* DH5α cells. Plasmids were isolated using the GeneJetTM Plasmid Miniprep Kit after manufacturers’ instructions (Fermentas, Heidelberg, Germany). BlastN search was used to identify the isolates on genus level at www.ncbi.nlm.nih.gov.

### Screening of Bacteria for Plant Growth Promotion Abilities

Selected bacteria were incubated for 2–3 days in liquid StdI at room temperature. Optical densities (OD_600_) of the cultures were measured and their supernatant used for Salkowski assay to determine IAA concentrations (Gordon and Weber, 1951). The ability to produce siderophores or mobilize phosphate were assessed on CAS (Schwyn and Neilands, 1987) or Pikovskaya agar medium (Mehta and Nautiyal, 2001) after 1 week of incubation at room temperature. The production of antibiotics was tested using the gram negative bacterium *Escherichia coli* and the gram positive *Micrococcus luteus*. They were grown by shaking in 30 ml liquid StdI medium for 12 h. Hundred microliter of both liquid cultures were transferred in 7 ml StdI with 7% agar, and transferred on plates with bacterial cultures to check their ability to produce antibiotics. All experiments were performed in triplicates and controls with non-inoculated plates were used. We additionally selected four bacteria (MRZ-1 through MRZ-4) based on their characteristics and investigated growth on nitrogen-free medium (5 g glucose, 5 g mannitol, 0.1 g CaCl_2_ × 2H_2_O, 5 mg Na_2_MoO_4_ × 2H_2_O, 0.9 g K_2_HPO_4_, 0.1 g KH_2_PO_4_, 0.01 g FeSO_4_ × H_2_O) for potential nitrogen fixation. These isolates (*Bacillus cereus* MRZ-1 (JMRC:ST:036355), *Lysinibacillus* sp. MRZ-2 (JMRC:ST:036356), *Bacillus pumilus* MRZ-3 (JMRC:ST:036358), and *Bacillus zhangzhouensis* MRZ-4 (JMRC:ST:036357) were deposited at Jena Microbial Resource Collection (JMRC), Jena, Germany.

### Effect of Selected Bacterial Isolates on *T. vaccinum* and/or *P. abies*

To evaluate the effects of isolated bacteria on *T. vaccinum* GK6514 (JMRC:FSU:4731, JMRC, Jena, Germany), 100 μl supernatant of bacterial overnight cultures (isolates MRZ-1 through MRZ-4, and pure StdI medium for control) grown in StdI (Merck, Darmstadt, Germany) were obtained and plated on modified Melin Norkrans b (MMNb) medium (Kottke et al., 1987), followed by *T. vaccinum* inoculation and cultivation over 4 weeks. The mycelial diameter and hyphal branching were recorded counting 100 hyphal tips selected randomly for branching within 500 μm distance from the tip. The influence of volatiles was examined using divided plates, where one half was inoculated with bacteria (four isolates: MRZ-1 through MRZ-4, and without inoculation for control) on StdI medium, while the other half contained 2 weeks old colonies of *T. vaccinum* on MMNb medium. After 20 days the evaluation of growth properties was performed using the software Spot version 4.6 (Diagnostic Instruments, Munich, Germany). Every treatment was performed in triplicates. The impact of the bacteria on germination of *Picea abies* seedlings (Landesforst Mecklenburg-Vorpommern, Germany) was investigated on germination medium (Krause and Kothe, 2006) using bacterial overnight cultures (set to OD_600_ = 0.1 after washing in 0.9% NaCl) and surface sterilized seeds, mixed for 1 min, and transferred to germination medium (ten seeds per plate). For control, treatment without bacteria was performed. All experiments were performed in triplicates. Data were recorded after 20 days.

For the investigation of the mycorrhization rate with selected bacteria, *P. abies* and *T. vaccinum* were cultivated in hydroponic cultures (Henke et al., 2015a) after axenic *P. abies* pre-cultivation on germination medium over 4 weeks and in the hydroponic system over 3 weeks before *T. vaccinum* was inoculated. After 3 more weeks, 100 μl of the selected bacteria with an OD_600_ of 0.1 washed in 0.9% NaCl were inoculated. After 3 months, the hydroponic cultures were evaluated for spruce vitality, root and shoot biomass, root architecture, fungal biomass and the mycorrhization rate of the root system in percentage of total roots with obvious mantle formation or mycorrhiza-typical thickened and coiled short root morphology.

### Isolation of Fungi With Selective Media

A soil dilution series (1 g soil 1:10 w/v in 0.9% NaCl) was plated on MMNb, well suited for ectomycorrhizal basidiomycetes, or supplemented minimal medium (SUP), optimal for the growth of mucoromycetes (Wöstemeyer, 1985). Spectinomycin or cycloheximide were added to prevent bacterial growth or inhibit most fungi to provide access to some resistant but less competitive genera like *Absidia*. Incubation at 22 or 28°C for 3–4 days or 4 weeks to select slow growing fungi was followed by selection of different OTUs and the total number of fungal colony forming units (cfu) was calculated. DNA was isolated from all OTUs with three freeze/thaw cycles of the mycelium in liquid nitrogen followed by heating for 1 min at 50°C followed by ITS PCR (primers ITS1: TCC GTA GGT GAA CCT GCG G and ITS 4: TCC TCC GCT TAT TGA TAT GC; see Innis et al., 1990) and identification was performed through sequencing (GATC Biotech, Germany).

### Microcosm Experiments

Microcosm tubes of 50 ml contained 20 ml sieved (<2 mm) soil with its natural microbial community. After treatment with 30% hydrogen peroxide for 1.5 h and rinsing with sterilized water *P. abies* seeds were placed on germination medium (Kottke et al., 1987). A 2 months sterily pre-grown *P. abies* seedling was planted using a sterile spatula and watered with sterile tap water. The microcosms were inoculated with *T. vaccinum* GK6514 and/or plus and minus mating types of the ubiquitous soil fungus *Mucor mucedo* (SF:JMRC:000620 or SF:JMRC:000621, JMRC, Jena, Germany). Strains of *Mucor* were chosen, because they may affect phytohormone signaling of fungi and trees and modulate their morphology (Wagner et al., 2016). Using a sterile stamp 7 mm diameter inocula of *T. vaccinum* were transferred from MMNb cultivation plates and for *M. mucedo* the same procedure was performed from SUP cultivation plates. The microcosms were incubated in a climate chamber (12 h light/23°C; 12 h dark/17°C; 80% humidity) and watered with 1 ml sterile tap water weekly (*n* = 12). After 8 weeks, vitality of trees (living or dead), microorganisms plated on SUP and MMNb for OTUs, and fungal CFU were checked.

### Effect of *T. vaccinum* on Spruce Pathogens

To analyze the role of *T. vaccinum* to protect the tree from pathogen attack *Botrytis cinerea* (SF:JMRC:001099) and *Heterobasidion annosum* S/F type (SF:JMRC:008560; both JRMC, Jena, Germany) were used. Well-overgrown agar blocks (5 mm × 5 mm) of both fungi were inoculated on MMNb plates with pre-grown (2 weeks) *T. vaccinum*, (see Supplementary Figure S3). After 2 weeks, pathogen growth was evaluated in comparison with pure cultures of the fungi using Image J 1.46^3^. The effect of volatiles of *T. vaccinum* on the phytopathogens was scored using divided plates with pure and co-cultures (see Supplementary Figure S3B). The disease symptoms of pathogens were checked with plate cultures (see Supplementary Figure S3C), where an axenic 8 weeks old spruce seedling was placed between two sterile cellophane membranes with fungal inocula on MMN agar media with 2% glucose and without malt extract (modified after Kottke et al., 1987). For quantitative data on pathogen protection, hydroponic cultures (Henke et al., 2015a) were used with the more aggressive and faster pathogen, *B. cinerea* (see Supplementary Figure S3D). Three 8 weeks old spruce seedlings were inoculated with two well-overgrown agar blocks of *T. vaccinum.* After 3 weeks *B. cinerea* was added (*n* = 4). Cultures containing either *T. vaccinum* or the pathogen were used as control. After 1 month, living trees were counted, root and shoot dried and biomass examined.

### *In silico* Analyses

The genome of *T. vaccinum* GK6514 was screened for genes involved in the biosynthesis of the pigment melanin, extending both to DHN melanin (Tsai et al., 1999) and L-DOPA melanin (Eisenman and Casadevall, 2012). The genome is available on request via JGI IMG^4^ under the submission ID59348. BLASTN search was performed with software sequenceserver^5^ and with NCBI database^6^.

### Statistical Analyses

Every treatment was performed in triplicates if not mentioned otherwise. The statistical analyses for comparison of two treatments was performed with unpaired Student’s *t*-test, and for more than two treatments with one-way analysis of variance (ANOVA) after Levene’s test. Tukey test was used for *post hoc* analyses. Significance levels are indicated with small letters and were set to *P* < 0.05. Data are shown as average values ± standard deviation.

## Results

### The Mycorrhizosphere Habitat

In order to establish potential interactions of fungi or bacteria present in the mycorrhizosphere surrounding *T. vaccinum* ectomycorrhiza, soil from the natural habitat was characterized. The soil samples were slightly acidic at pH 6.5 and featured high concentrations of bioavailable lanthanides, Pb and Mn (see Supplementary Table S1). A high C (26.92% ± 0.012) to N (0.84% ± 0.008) ratio of 32:1 and raised S contents (0.11% ± 0.009) were observed.

**FIGURE 1:**
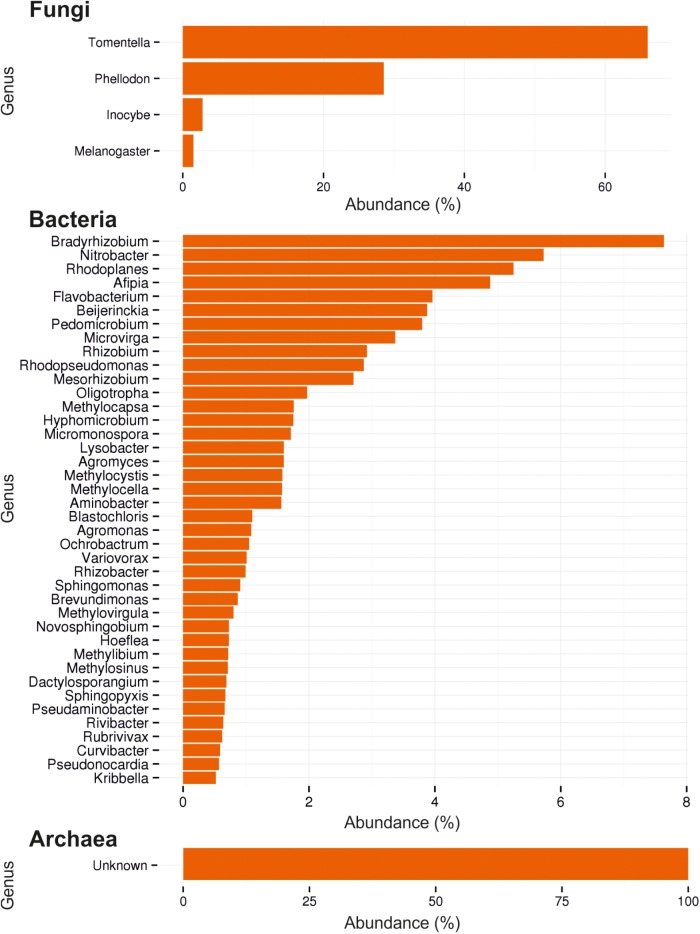
Ectomycorrhizosphere community assessment by ITS and 16S rDNA sequencing. Composition of fungi, bacteria and archaea by most abundant reads (%) are shown.

In soil from O, L, and H horizon of this forest habitat, the fungal community composition was determined (Figure 1). For fungi it revealed 53 OTUs consisting mainly of Basidiomycota (99.95%), with almost 95% of the basidiomycetes being ectomycorrhizal members of *Thelephorales* and 3.5% *Agaricales* (Table 1). Among the most abundant genera, 16 basidiomycetes, 12 with an ectomycorrhizal lifestyle, were identified. At species level, the dominant species clearly was *Tomentella lateritia*.

**Table 1:** Identified OTUs by pyrosequencing (90% sequence similarity level) and their different lifestyles.

Clade	Life style^∗^
**ASCOMYCOTA**	
**Sordariomycetes**	
**Hypocreales**	
*Hypocrea_stellata*	MPAR
*Trichoderma_*sp.	MPAR
*Hypocrea_cremea*	MPAR
*Hypocrea_chlorospora*	MPAR
**BASIDIOMYCOTA**	
**Agaricomycetes**	
**Agaricales**	
*Alnicola_*sp.	ECM
*Cortinarius_infractus*	ECM
*Cortinarius_cinnamomeus*	ECM
*Cortinarius_cedretorum*	ECM
*Cortinarius_cupreorufus*	ECM
*Cortinarius_*sp.	ECM
*Cortinarius_*sp.	ECM
*Cortinarius_langeorum*	ECM
*Cortinarius_aurora*	ECM
*Cortinarius_caesibulga*	ECM
*Cortinarius_cinereoroseolus*	ECM
*Cortinarius_argyronius*	ECM
*Cortinarius_eutactus*	ECM
*Inocybe_dulcamara*	ECM
*Inocybe_substraminipes*	ECM
*Inocybe_substraminipes*	ECM
*Inocybe_terrigena*	ECM
*Inocybe_leucoblema*	ECM
*Inocybe_leucoloma*	ECM
*Inocybe_myriadophylla*	ECM
*Inocybe_cf_dulcamara*	ECM
*Hebeloma_testaceum*	ECM
*Hebeloma_syrjense*	ECM
*Hymenogaster_luteus_var._luteus*	ECM
*Lepiota_acutesquamosa*	SAP
*Lyophyllum_shimeji*	ECM
*Stropharia_inuncta*	SAP
*Stropharia_hornemannii*	SAP
*Tricholoma_ustale*	ECM
*Tricholoma_psammopus*	ECM
*Tricholoma_*sp.	ECM
**Boletales**	
*Melanogaster_broomeianus*	ECM
*Melanogaster_variegatus*	ECM
**Polyporales**	
*Perenniporia_pyricola*	MPAR
*Perenniporia_truncatospora*	PPATH
**Russulales**	
*Russula_aquosa*	ECM
*Lactarius_blennius*	ECM
**Thelephorales**	
*Tomentella_lateritia*	ECM

*Tomentella_cinerascens*	ECM
*Tomentella_*sp.	ECM
*Pseudotomentella_*sp.	ECM
*Phellodon*_sp.	ECM


**^∗^ ECM, ectomycorrhizal; PPATH, phytopathogenic; MPAR, mycoparasitic; SAP, saprophytic.**

**FIGURE 2:**
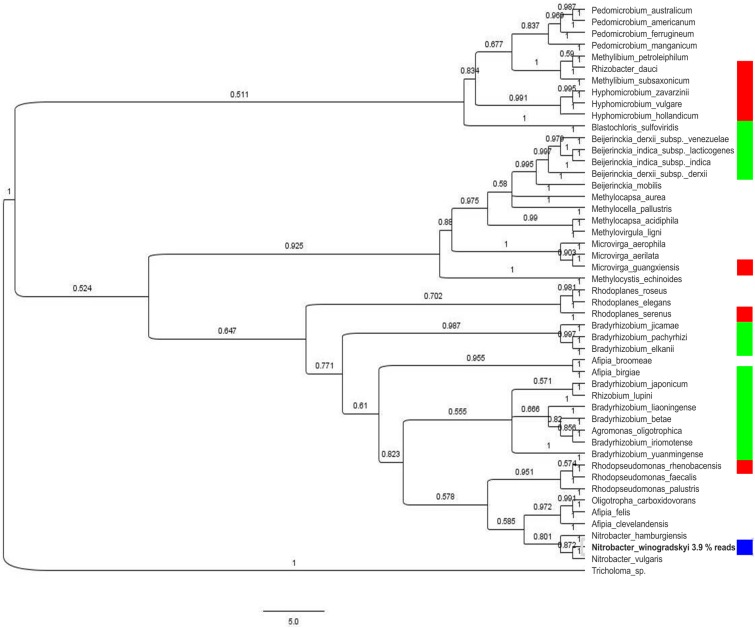
Phylogenetic tree of the 16S rDNA fragments of the most abundant species (over 0.5% composition reads) on 93% sequence similarity level. Relevance in nitrogen cycling is indicated for nitrogen fixation (green), nitrification (blue), and denitrification (red).

The bacterial community showed a high diversity with 1517 different bacterial OTUs detected (Supplementary Table S3). The five most abundant genera were *Rhodoplanes* (19.5%), *Pedomicrobium* (8.16%), *Afipia* (6.93%), *Methylibium* (5.02%), and *Dongia* (4.67%), the three dominant ones being *Rhizobiales* (compare Figure 1). On species level, the mycorrhizosphere was dominated by the *Rhizobiales* member *Nitrobacter winogradskyi* with 3.9% of reads, with another 0.5% being other *Rhizobiales* with potential function in nitrogen cycling (Figure 2).

Of the detected OTUs, 505 represented less than 0.01% of the total community (<18 reads), containing mainly Gram-positives with high species heterogeneity (44% Actinobacteria). Among the archaea, low abundance and low diversity, again hinting at nitrogen cycling, was detected.

### Physiological Traits of Isolated Fungi and Bacteria

To assess the physiological potential of strains present in the mycorrhizosphere, fungal and bacterial isolates were obtained. On complex media the conidiospore forming genus *Penicillium* dominated. At lower temperature and with increased incubation time, fungi with different life styles were isolated (Table 2). Using antibiotics, different taxa were enriched with cycloheximide selecting for *Absidia* mucoromycetes and ascomycetous *Hypocreales*, namely *Lecanicillum*, *Acremonium*, *Beauveria*, and *Penicillium* (see Table 2), while spectinomycin led to *Penicillium* and *Beauveria* isolation, with the Hypocreales fungus *Pochonia* and the Mucoromycota *Mortierella* and *Umbelopsis* being enriched. Of all fungal isolates, only 6.3% produced IAA without tryptophan addition, and 7.4% with the addition of 0.5 mM tryptophan to the medium.

**Table 2:** Isolated fungi and their different life styles.

Clade	Lifestyle^∗^	Isolation from soil
**BASIDIOMYCOTA**		
*Tricholoma*	ECM	+
*Hypholoma*	SAP	+
*Kuehneromyces*	SAP	+ (2 strains)
*Psathyrella*	SAP	+ (3 strains)
**ASCOMYCOTA**		
*Hypocrea/Hypocreales*	MPAR	+
*Penicillium*	SAP, PATH	+ (24 strains)
*Beauveria*	EPATH	+ (4 strains)
*Acremonium*	SAP, PATH	+
*Lecanicillium*	EPATH	+ (2 strains)
*Cryptosporiopsis*	PPATH	+
*Microdochium*	PPATH	+ (2 strains)
*Helotiales*	PPATH, MYC, SAP, MPAR	+ (2 strains)
*Cladosporium*	SAP	+
*Mycosphearella*	PPATH	+
**MUCOROMYCOTA**		
*Umbelopsis*		+ (5 strains)
*Mortierella*	SAP	+ (4 strains)
*Absidia*	PATH	+ (3 strains)


**^∗^ ECM, ectomycorrhizal; PPATH, phytopathogenic; MPAR, mycoparasitic; EPATH, entomopathogenic; PATH, pathogenic for mammals; SAP, saprophytic.**

**FIGURE 3:**
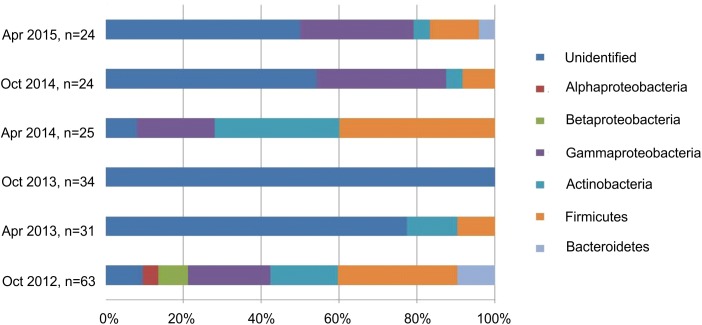
Composition of the isolated bacteria (%) at different sampling times.

To detect seasonal changes in the mycorrhizosphere community and the related changes in ecological functions relating to the formation of mycorrhiza and tree growth, bacteria were isolated in three different years from the same site during spring and autumn (Figure 3). The average bacterial abundance was found to be around 10^7^ with 111 isolated strains belonging to *Bacillus* (*n* = 29), *Pseudomonas* (*n* = 27), *Micrococcus* (*n* = 11), *Streptomyces* (*n* = 6), *Bacteroides* and α- and ß-proteobacteria including *Burkholderia* (*n* = 2). The selected isolates could be shown to provide a range of plant growth promoting abilities with changes visible specifically for siderophore production between different time points and seasons (Supplementary Figure S4). Around 23% of the isolates produced siderophores, 22% mobilized phosphate, and around 74% excreted the phytohormone IAA. Streptomycetes, *Micrococcus* and *Pseudomonas* showed the highest potential to mobilize phosphate, and all of the dominant phyla produced IAA (Supplementary Table S3).

### *T. vaccinum* Alters the Fungal Community Structure in Microcosms

To see the influence of *T. vaccinum* on fungal community and spruce health, we used microcosms with soil obtained from the habitat. After 8 weeks of cultivation, the transplanted spruce seedlings showed a high mortality. A tendency to a higher vitality with *T. vaccinum* (58% alive compared to 25% in control) was visible. Inoculation with the non-wood degrading mucoromycete *M. mucedo* slightly increased spruce health (42% alive). No significant difference of shoot, primary and lateral root growth, or needle development was obtained at the seedling stage (data not shown).

**FIGURE 4:**
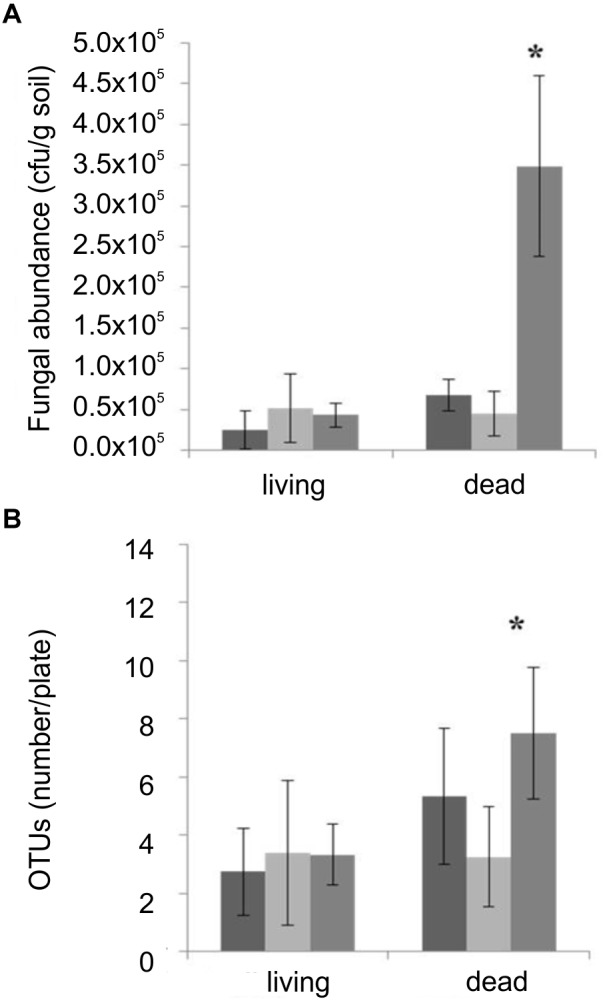
Fungal CFU **(A)** and OTUs **(B)** compared between microcosms non-inoculated (black), inoculated with *M. mucedo* (light gray) or *T. vaccinum* (dark gray) of living or dead spruce trees. ^∗^Significance level, *p* < 0.05, error bars indicate standard deviation; *n* = 12.

An effect of *T. vaccinum* on the mycorrhizosphere was obtained in the microcosms containing dead trees. There, *T. vaccinum* significantly increased the number of fungal colonies and OTUs indicating a higher prevalence of plant decomposers, if *T. vaccinum* was present (Figure 4).

To show the ability of *T. vaccinum* to degrade plant material, it was grown on MMNb where the only C source was replaced by cellulose, the main component of plant cell walls, which supported growth (Supplementary Figure S5).

### Mycorrhiza Helper Bacteria

Four isolates were selected based on their ability to produce IAA, siderophores, or to grow on nitrogen-free medium suggesting nitrogen fixation (Supplementary Tables S3, S4). *Bacillus cereus* MRZ-1, *Lysinibacillus* sp. MRZ-2, *Bacillus pumilus* MRZ-3 and *Bacillus zhangzhouensis* MRZ-4 all synthesized IAA, but did not solubilize phosphate; *B. cereus* MRZ-1 produced IAA only in the presence of an additional tryptophan source. All, with the exception of *Lysinibacillus* sp. MRZ-2, grew on nitrogen-free medium. These were tested for their effect on *Tricholoma* and mycorrhiza.

**FIGURE 5:**
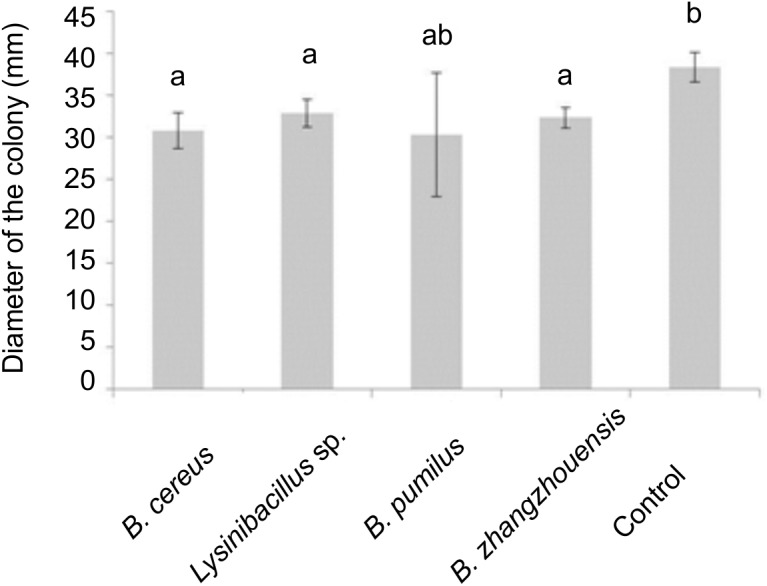
Influence of bacterial volatiles in divided plates on the diameter of the colony of *T. vaccinum*, significance level *p* < 0.05, *n* = 3.

Volatile compounds produced by bacteria *B. cereus* MRZ-1, *Lysinibacillus* sp. MRZ-2, and *B. zhangzhouensis* MRZ-4 affected fungal growth and significantly reduced the colony diameter of axenically grown *T. vaccinum* (Figure 5), when co-cultivated on divided plates that allow for volatile transmission, but not for diffusion of compounds. Volatiles, especially of *B. cereus* MRZ-1, strongly induced the formation of a brownish pigment by *T. vaccinum* which was not observed using bacterial supernatant (Supplementary Figure S6). To see whether this pigment might be melanin, the genome of *T. vaccinum* was checked for genes involved in melanin biosynthesis. Genes *alb1* (ID: g2107.t1), *arp1* (g3608.t1), *arp2* (g10130.t1), *abr1* (g4688.t1), and *abr2* (g3608.t1), known to be involved in the biosynthesis of DHN melanin, as well as the laccase *lac1* (g4688.t1) of L-DOPA melanin biosynthesis could be identified.

In addition to volatiles, supernatants were checked for morphological effects. *B. cereus* MRZ-1 sterile culture filtrate increased primary and secondary hyphal branching of *T. vaccinum* (number of 1st order branches increased from 1.76 to 2.95 and 2nd order branches from 1.01 to 2.83), which was not the case with the other bacteria tested.

**FIGURE 6:**
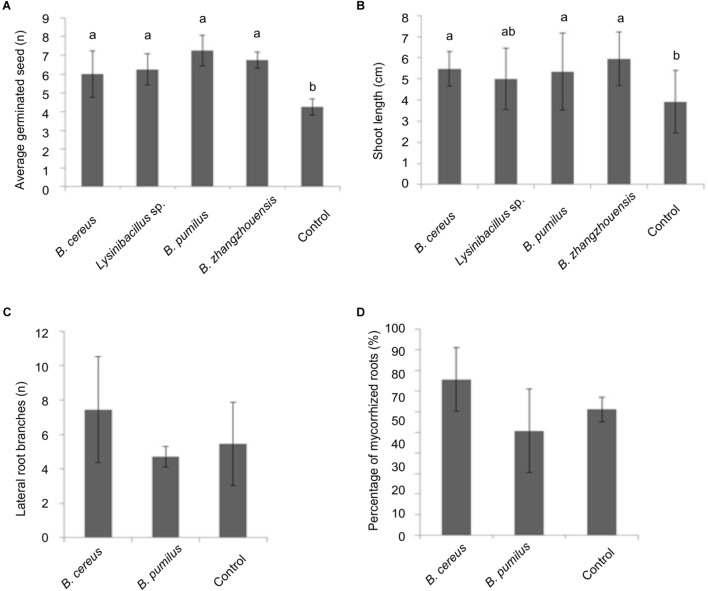
Effect of suspension of the selected bacterial isolates on *P. abies* **(A)** germination and **(B)** germination tube or shoot length, significance level *p* < 0.05. Effect of bacterial suspension on mycorrhized trees after 3 months of incubation with total number of lateral branches per root system **(C)** or percentage of total mycorrhized roots **(D)**, error bars indicate standard deviations, *n* = 3.

As for an impact on tree development, germination rate of spruce seedlings inoculated with the bacterial suspension showed a slight increase (Figure 6). A significantly higher number of germinated seeds had an elongated germination tube after 20 days (not shown). Moreover, the length of the germination tubes that sometimes already developed into a shoot was significantly enhanced when treated with *B. cereus* MRZ-1, *B. pumilus* MRZ-3, and *B. zhangzhouensis* MRZ-4 (see Figure 6B).

Suspension of the two isolates with best effects were then screened for their impact on mycorrhization. After 3 months, mycorrhiza formation was slightly improved by *B. cereus* MRZ-1 with increased number of lateral branches in the root system (see Figure 6C) and mycorrhization rates (see Figure 6D).

### *T. vaccinum* Reduced Pathogen Growth

The protection of mycorrhiza against pathogen attack was shown using *T. vaccinum*, which significantly reduced the growth of both pathogens tested *in vitro* (Supplementary Figure S7).

The reduced growth of the root pathogen *H. annosum* already allows for some protection of the host tree. In this case, volatiles emitted by *T. vaccinum* had no effect on *H. annosum* in divided plates.

For the more severe and faster growing needle pathogen *B. cinerea*, a slight effect of volatiles was seen in addition to growth reduction in co-culture indicating the potential to work at a distance (see Supplementary Figure S7).

**FIGURE 7:**
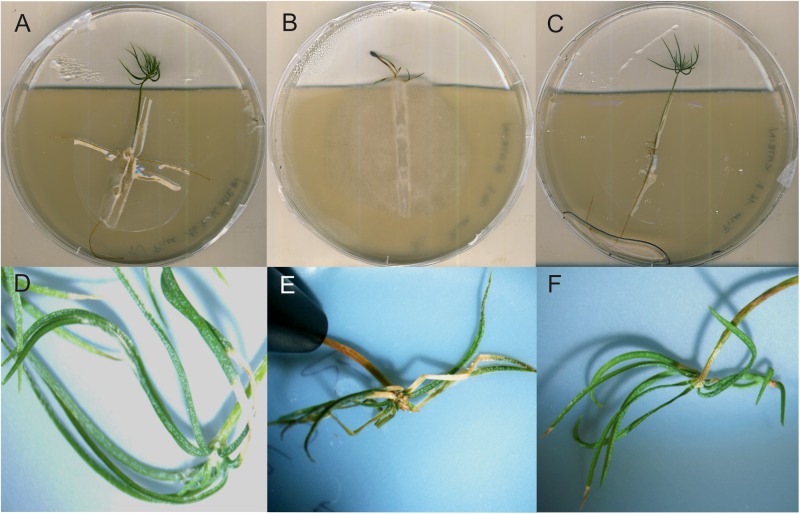
Spruce seedlings inoculated with *T. vaccinum* **(A,D)**, with *B. cinerea* displaying typical symptoms in needle development **(B,E)**, co-inoculated, healthy seedlings **(F)**, and without fungal inoculation **(C)**.

In hydroponic mycorrhizal co-cultures, *T. vaccinum* redirected the growth of *B. cinerea* to the medium-air interface, away from the *T. vaccinum* inoculant. From 12 incubated spruces, all survived when inoculated with *T. vaccinum* or in the co-inoculation of *T. vaccinum* and *B. cinerea*. All spruces of both treatments appeared healthy, whereas only 9 of 12 spruces that were incubated with the pathogen alone were alive. The pathogen stayed at the root and caused typical disease symptoms like needle discoloration, needle death or black points at needles (Figure 7).

## Discussion

Here, we analyzed the composition of the microbial community in a spruce mycorrhizosphere and the effect of the ectomycorrhizal fungus *T. vaccinum* on community structure in a microcosm experiment. Pyrosequencing revealed a high diversity in Basidiomycota, whereas Ascomycota were underrepresented, and other phyla did not appear. Ten most abundant OTUs featured an ECM lifestyle, and clearly reflected the fact that forest trees are colonized by different ECM fungi simultaneously (Smith and Read, 1987). We also could show a much higher diversity at the sampling site than had been reported earlier for the ECM community of Norway spruce with 34 different taxa, which is known to change between tree species, geographical as well as climatic conditions, soil composition and seasons (Dighton et al., 2005; Korkama et al., 2006). Thus, a saturated diversity sampling seems to be highly important, which was checked in this study by rarefaction analysis (compare Supplementary Figure S2). Remarkably, the 10 most abundant OTUs from this work were not represented in the *P. abies* ECM study performed by Korkama et al. (2006) indicating the high variability between habitats.

We isolated common soil colonizing and fast growing fungi, which might have outcompeted in culture other slower growing fungi detected in amplicon sequencing. The isolates belong to the genera *Umbelopsis*, *Penicillium*, *Mortierella*, *Absidia* (Warcup, 1951), insect or nematode parasites *Beauveria* (Meyling and Eilenberg, 2006) and *Pochonia* (Atkins et al., 2003). *Umbelopsis ramanniana*, different *Penicillium* species, *Beauveria bassiana* and *Pochonia bulbillosa* were frequently found in free soil, the rhizosphere, mycorrhizosphere and hyphosphere of spruce trees (Voronina, 2011). Additionally, *Penicillium* and *Umbelopsis* species have been described as endophytes in spruce roots (Kernaghan and Patriquin, 2011). Species of *Lecanicillium* (Goettel et al., 2008) and *Acremonium* (Latch, 1994) can colonize grasses as endophytes, but were also found in the rhizosphere of healthy black spruce trees (Vujanovic et al., 2007) and *Lecanicillium* sp. in the hyphosphere (Voronina, 2011).

Voronina (2011) showed increased mycomycete diversity in *T. fulvum* mycorrhizosphere compared to the hyphosphere alone, which was not the case for most of the other studied ECM fungi. These experiments fit well with the microcosm studies, where a higher abundance and diversity of fungi was observed with dead spruce trees that might provide a saprotrophic food source. Since *T. vaccinum* is able to use cellulose as sole C source, its degradative capacity could possibly have supported the growth of other fungi through decomposition of dead spruce trees. The ability of ECM fungi to live as saprotrophs which can decompose plant litter is intensively discussed (Baldrian, 2009). The sequenced genome of *T. vaccinum* covers multiple gene copies coding for cellulolytic enzymes and laccases – more than the ECM average (Wagner et al., 2015). Interestingly, its activity decreased in the presence of spruce root exudates, functioning as early signaling molecules (Wagner et al., 2015). For other ECM fungi, decomposition of proteins, cellulose, hemicellulose and other organic carbons was shown, as well as the colonization of soil organic matter patches (Haselwandter et al., 1990; Bending and Read, 1995).

The high C:N ratio detected at the test site suggests that the studied mycorrhizosphere is a nitrogen limited habitat with a potentially reduced microbial activity (Paul, 2014). Research pointed out that nitrogen fixation is essential in mycorrhizal interactions and raised the question if the symbiotic fungal partners can perform this task, or if this function is delegated to MHB (Mikola, 1986). This hypothesis was strengthened by detection of nitrogen fixing *Bradyrhizobium japonicum* on non-legumes, as is seen with the present study (Lian et al., 2002). Further, three of four proposed MHB showed growth on nitrogen-free medium suggesting nitrogen fixation (compare Supplementary Table S4).

ECM microbial communities are diverse, but share structural similarities not present with the surrounding bulk soil. Uroz et al. (2012) identified Proteobacteria with 56%; the 10 dominant genera were *Acidobacteria* (19%), *Burkholderia* (6%; with a function in mineral weathering), *Rhodoplanes* (5%), *Chitinophage* (4%), and *Bradyrhizobium* (4%) in the mycorrhizosphere of oak with *Scleroderma citrinum* and *Xerocomus pruinatus*. Here Alphaproteobacteria dominated the mycorrhizophere, including two *Bradyrhizobium* species (together 2.27%) and *Rhodoplanes* (together 4.24%).

A largely dissimilar microbial diversity was observed by analysis of cultivated microbial isolates, showing the bias of cultivation versus different recovery rates during DNA isolation, amplification and sequencing. Our isolation supported fast growers and omitted obligate symbionts and other bacteria with special growth requirements (compare Bakken, 1997). We isolated mostly streptomycetes, pseudomonads, and *Bacillus* or *Micrococcus* species, indicating the higher abundance of Gram-positives and pseudomonads compared to the pyrosequencing result. Sixty *Pseudomonas fluorescens* strains had been isolated from a mycorrhizosphere including 81% inorganic phosphate mobilizers, 80% IAA and 74% siderophore producers (Frey-Klett et al., 2005). These values are higher compared to our results, which might be attributed to our broader selection for all bacterial phyla with, e.g., actinobacteria that have been shown to impact the symbiosis positively, like the positive effect reported for *Streptomyces orientalis* on spore germination of *Glomus mosseae* (Mugnier and Mosse, 1987).

The majority of the isolated bacteria showed plant growth promoting abilities; especially auxin biosynthesis was found with most strains. IAA is able to increase branching of *T. vaccinum* and increases mycorrhization with *P. abies* (Krause et al., 2015). Thus, hyperbranching in the presence of *B. cereus* MRZ-1 might be related to IAA produced only when tryptophan from root exudates induces IAA synthesis (Frey-Klett et al., 2005). Changes in the IAA flux, induced by morphogenic compounds in the mycorrhizosphere like the fungal intermediate D’orenone, also may alter the physiology of the symbiotic partners and ECM formation (Wagner et al., 2015). Moreover, all four MHB produced IAA and stimulated the germination and shoot growth in *P. abies*. Siderophores produced by three of the selected bacteria (*B. cereus* MRZ-1, *B. pumilus* MRZ-3, *B. zhangzhouensis* MRZ-4) may be involved in shoot elongation seen with MHB through enhanced iron bioavailability in addition to siderophores from ECM and AM fungi reported to increase iron uptake in host plants (Haselwandter, 1995). The siderophore production and phosphate mobilization ability varied between our samples, possibly caused by a seasonal effect, abiotic factors like water availability, or heterogeneity in the sampling microhabitat.

*B. cereus* MRZ-1 showed the highest potential as MHB because it positively affected spruce germination and shoot growth, enhanced lateral branching and the rate of mycorrhization and spruce health. Moreover, volatiles changed *T. vaccinum* morphology by increasing its pigment production and reducing the mycelia diameter. We interpret this feature of a somewhat decreased extraradical growth not detrimental to advancing mycorrhization, since an earlier and more pronounced formation of intraradical symbiotic structures might be the result of this interaction. This warrants further research into signaling via volatile compounds. The pigment production might be linked to melanin, reported to mediate fungal stress resistance toward radiation, drought or reactive oxygen species, as well as being a virulence factor for plant and human pathogens (Pagano and Dhar, 2015). The initiation of pigment production in *T. vaccinum* thus could be a stress response induced through bacterial volatiles, and subsequently increase fungal vitality and thus mycorrhization.

*T. vaccinum* enhanced the survival rate of spruce trees during 8 weeks of incubation, which is impressive because mycorrhization evolves over several months. The positive effect on the plant partner in early mycorrhization has been discussed to involve stimulation of defense mechanisms with phytohormone signaling, because *T. vaccinum* is able to produce jasmonic acid, ethylene and salicylic acid (Wagner, 2016), with jasmonic and salicylic acids known to increase spruce defense against the stem and butt rot causing fungus *H. parviporum* (Arnerup et al., 2013). While the related *H. annosum* that infects spruce *via* the root (Stenlid and Johansson, 1987) did not respond to volatiles produced by *T. vaccinum*, the pathogen *B. cinerea* was impaired in growth. The induction of systemic acquired resistance through salicylic acid production by *T. vaccinum* can confer protection even against a needle pathogen such as *B. cinerea*. In addition, volatiles of *T. vaccinum* included potential antimicrobial compounds, like the typical fungal volatiles octen3-ol and 3-octanone, the terpenoid plant metabolites limonene and β- barbatene, as well as geosmin (Wagner, 2016). The mycorrhization with *T. vaccinum*, at the same time, changed the VOC pattern of spruce. Increased limonene production with antimicrobial activity (Henke et al., 2015b) may also have contributed to resistance against *B. cinerea* root infection. Thus, further research on the precise identification of the chemical components of the volatiles involved in the specific interaction is needed.

With the holistic approach on the entire mycorrhizosphere, we could show complex interactions involving more than two partners. *T. vaccinum*, as ectomycorrhizal fungus, enhances survival of its host, spruce. At the same time, *T. vaccinum* can suppress pathogen attack likely by induction of plant defense and antimicrobial activity of *T. vaccinum* compounds. A third function of *T. vaccinum* is correlated with its saprotrophic growth, where it decomposed dead host material and, thus, changed the fungal community present in the mycorrhizosphere. Bacteria isolated from mycorrhizspheric soil were shown to act as MHB through the production of phytohormones and volatiles, which might be true as well for other, non-cultivable members of the bacterial community.

## Author Contributions

KW designed and conducted experiments, analyzed, and wrote the first draft of the manuscript. DS, RG-M, and KK conducted experiments, analyzed, and contributed to the manuscript preparation. AK, KK, and EK designed experiments, interpreted results, and contributed to the manuscript preparation.

## Conflict of Interest Statement

The authors declare that the research was conducted in the absence of any commercial or financial relationships that could be construed as a potential conflict of interest.

## Footnotes

1 http://mafft.cbrc.jp/alignment/server/

2 http://mrbayes.sourceforge.net/

3 http://rsb.info.nih.gov/ij/download.html

4 http://img.jgi.doe.gov/

5 http://www.sequenceserver.com

6 http://blast.ncbi.nlm.nih.gov
