# Supplementary material for: The Ectomycorrhizospheric Habitat of Norway Spruce and Tricholoma vaccinum: Promotion of Plant Growth and Fitness by a Rich Microorganismic Community
Source: Front Microbiol. 2019 Feb 20;10:307. doi: 10.3389/fmicb.2019.00307 (PMC6391851; doi:10.3389/fmicb.2019.00307)
Supplement: Supplementary file 1 [file Data_Sheet_1.PDF]

Supplementary Material

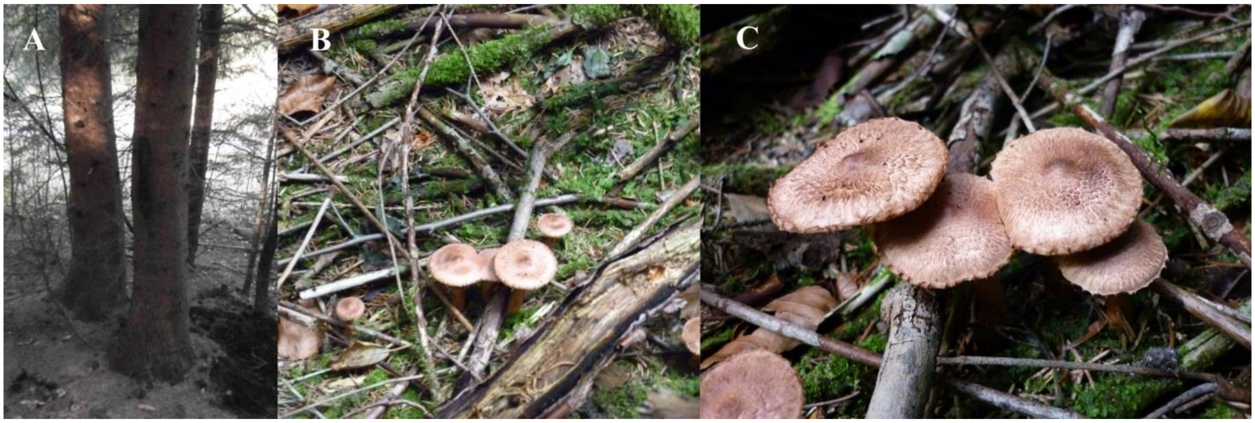

Figure S1: The sampling was performed in the rhizosphere of two spruce trees at a site characterized by old spruces and pines. The soil samples were taken at spruces with the next pine in a distance of more than 7 meters. (A). Fruiting bodies of *Tricholoma vaccinum* were found between *Sphagnum* mosses in October 2012 (B). They show the typical hairy reddish-brown caps (C).

Table S1: Sequential extraction of the mobile fraction F1 and the bioavailable fraction F2 in the mycorrhizosphere of *Picea abies* and *T. vaccinum* (ECM) compared to compost soil and, as a reference, contaminated soil from the former uranium mining area in Ronneburg, Germany (Schütze et al., 2014).

	F1 ECM	F1 compost	F1 reference	F2 ECM	F2 compost	F2 reference
**S**	44.1 ±2.112	190 ± 2	183 ± 3	19 ± 0	35.6 ± 0.4	161 ± 11
**Si**	288.7 ± 100.8	41 ± 0.5	38 ± 2	211.4 ± 46.5	16.5 ± 0.1	68.3 ± 0.8
**Ba**	53.4 ± 15.6	24.8 ± 0.1	128 ± 1	25.9 ± 11.5	10.7 ±0.1	57 ± 0.6
**Sr**	84.7 ± 8.9	41.7 ± 0.2	14.4 ± 0.1	84.3 ± 5.0	14.1 ± 0.1	3.5 ± 0.1
**P**	20.6 ± 0.7	174.7 ± 0.2	0 ± 0	15.7 ± 1.5	130 ± 1	n.d.
**Li**	0.2 ± 0.02	0.18 ± 0.002	0.09 ± 0.003	0.111 ± 0.028	0.05 ± 0.004	n.d.
**Ni**	0.097 ± 0.021	0.16 ± 0.01	3.2 ± 0.05	0 ± 0	n.d.	6.6 ± 0.3
**Cs**	0.02 ± 0.004	0.01 ± 0.002	0.2 ± 0.005	0.01 ± 0	n.d.	0.02 ± 0
**Pb**	0.1 ± 0.001	0.04 ± 0.001	n.d.	1.78 ± 0.01	0.29 ± 0.01	0.8 ± 0.001
**As**	0.04 ± 0.02	0.95 ± 0.05	n.d.	0.1 ± 0	0.34 ± 0.07	0.1 ± 0.02
**Cd**	0.02 ± 0.01	n.d.	0.3 ± 0.05	0.14 ± 0.05	0.01 ± 0.003	1.5 ± 0.01
**Co**	0.133 ± 0.025	0.075 ±0.002	0.4 ± 0.01	0.16 ± 0.04	0.06 ± 0	0.8 ± 0.02
**K**	660.4 ± 83.7	1770.3 ± 0.7	49.9 ± 0.2	89.8 ± 13.7	265.4 ±0.7	n.d.
**Mg**	322.6 ± 28.3	667 ± 5	325 ± 3	224.5 ± 12.28	219 ± 1	32.3 ± 0.4
**Mn**	56.7 ± 6.1	24.4 ± 0.1	28.8 ± 0.3	153.4 ± 21.6	21.8 ± 0.1	30.8 ± 0.4
**Na**	25.8 ± 3	192.9 ± 0.1	21.8 ± 0.5	15.3 ± 0.4	29.3 ± 0.1	n.d.
**Ca**	13132.7 ± 6.5	11717 ±50	1730 ± 0.3	22232.7 ± 1286	4438 ± 26	312 ± 4
**Al**	2.2 ± 0.28	2.4 ± 0.1	0.3 ± 0.05	1.5 ± 0.5	2.2 ± 0.04	7.9 ± 0.2
**Fe**	5.5 ± 1.1	4.7 ± 0.2	0.2 ± 0.01	3.13 ± 0.6	4.7 ± 0.3	0.9± 0.01
**Zn**	1.7 ± 0.4	0.73 ± 0.05	7.8± 0.1	6.1 ± 2.3	1.4 ± 0.2	33.7± 0.1
**Cu**	0.2 ± 0.07	0.002	4.1 ± 0.01	0.06 ± 0.018	0.08 ± 0.001	83.7 ± 0.2

n.d., not detected

Table S2: Read statistics and OTU assignment of pyrosequencing of fungal ITS1 region and 16S rRNA.

Description	Read pairs	% Read pairs	Read pairs	% Read pairs
**Read statistics of fungal ITS1 region**			**Read statistics of bacterial 16S rRNA**	
Total	368173	100.0	405249	100.0
Cleaned	368163	100.0	380065	93.8
Cleaned (orphan)	10	0.0	24438	6.0
Merged by overlapping	233292	63.4	310444	76.6
Clustered by similarity	227803	61.9	247281	61.0
Chimeric	0	0.0	0	0.0
Final high quality	227803	61.9	247281	61.0
**OTU assignment of fungal ITS1 region**			**OTU assignment of bacterial 16S rRNA**	
High quality	227,803	100.0	247,281	100.0
OTU assigned	227,742	100.0	246,857	99.8
Filter passed OTUs	30,137	13.2	160,799	65.0

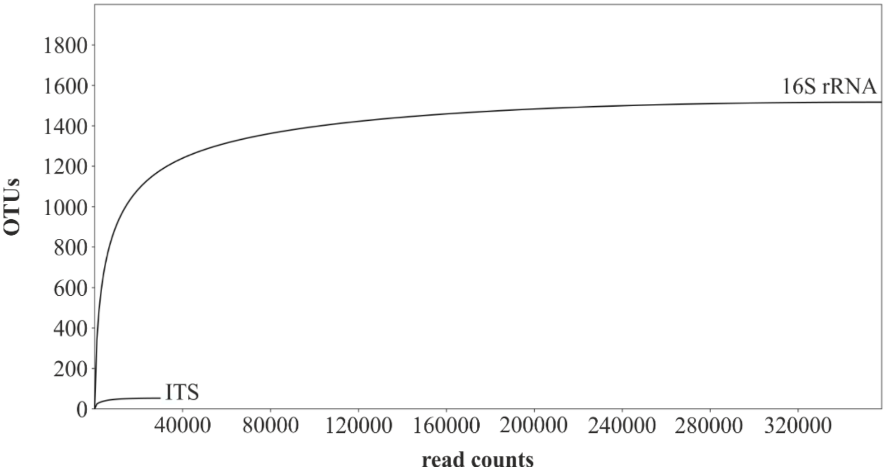

Figure S2: Rarefaction analysis of fungal ITS and bacterial 16S rRNA samples.

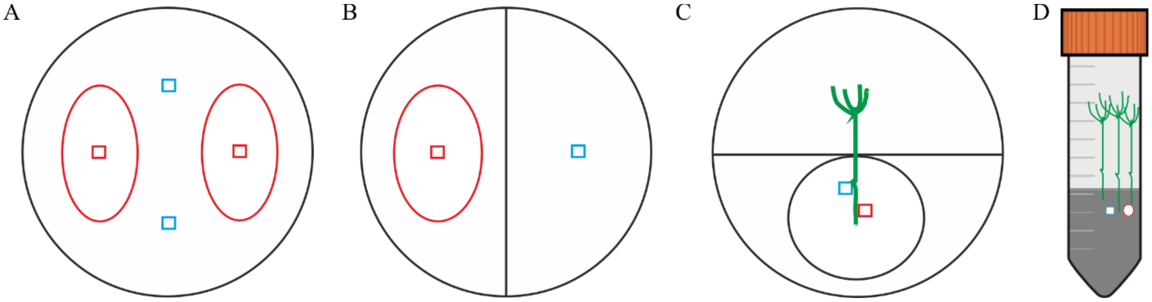

Figure S3: Four experimental setups were used to show the effect of *T. vaccinum* on spruce pathogens. Petri dish with pre-grown *T. vaccinum* on MMNb agar was inoculated with a pathogen (A), divided Petri dishes were inoculated with the pathogen to investigate the effect of volatiles (B), Petri dishes (C) and hydroponics with MMNa medium, spruce seedling(s), and *T.*

vaccinum were used; the pathogen was inoculated two weeks later (D). *T. vaccinum* (red), pathogen (blue).

Table S3: Bacterial isolates with their plant growth promoting abilities.

Genus	Sidero-phore production	Phos-phate mobili-zation	IAA (µM/bio-mass)	Genus	Sidero-phore production	Phos-phate mobili-zation	IAA (µM/bio-mass)
**Oct-2012**				**Apr-2013**			
Serratia	no	yes	no	*Aeromonas*	no	no	n.d.*
Micrococcus	no	no	no	unknown	no	no	n.d.
Pseudomonas	no	no	n.d.	unknown	n.d.	no	n.d.
Bacillus	no	no	n.d.	*Micrococcus*	n.d.	n.d.	n.d.
Variovorax	n.d.	n.d.	n.d.	unknown	yes	yes	n.d.
unknown	n.d.	n.d.	n.d.	unknown	n.d.	no	no
Pseudomonas	no	n.d.	n.d.	unknown	n.d.	yes	no
Pseudomonas	n.d.	n.d.	n.d.	unknown	n.d.	no	no
Erwinia	no	no	n.d.	unknown	no	yes	1.57
Bacteroidetes	yes	no	yes	unknown	no	no	2.89
Sphingobacterium	n.d.	n.d.	n.d.	unknown	no	no	0.60
Burkholderia	yes	n.d.	yes	unknown	no	n.d.	57.70
Bacillus	n.d.	n.d.	no	unknown	no	n.d.	52.6.
Bacillus	yes	no	no	unknown	no	n.d.	180.77
unknown	n.d.	n.d.	no	unknown	n.d.	n.d.	n.d.
Frigoribacterium	no	no	n.d.	unknown	no	no	2.36
Bacillus	n.d.	n.d.	yes	unknown	no	no	1.20
Bacillus	no	n.d.	yes	unknown	no	no	no
Sphingobacterium	no	n.d.	n.d.	*Streptomyces*	n.d.	no	3.95
Pseudomonas	no	no	n.d.	*Bacillus*	n.d.	no	no
Bacillus	n.d.	n.d.	no	*Bacillus*	n.d.	no	no
Bacillus	n.d.	n.d.	no	unknown	n.d.	yes	8.56
Pedobacter	n.d.	n.d.	yes	unknown	no	no	4.59
Streptomyces	no	yes	yes	unknown	no	yes	12.05
Paenibacillus	n.d.	n.d.	no	unknown	no	no	7.27
unknown	n.d.	n.d.	yes	unknown	no	no	11.67
Bacillus	n.d.	n.d.	n.d.	*Streptomyces*	no	yes	20.00
unknown	n.d.	n.d.	no	*Micrococcus*	no	yes	8.33
Pseudomonas	no	n.d.	n.d.	*Bacillus*	no	yes	91.98
Pedobacter	n.d.	n.d.	no	unknown	no	no	111.28
Pseudomonas	no	no	n.d.	unknown	no	no	192.86
Streptomyces	n.d.	n.d.	no				
Bacillus	n.d.	n.d.	n.d.				
Streptomyces	no	n.d.	no				
Burkolderia	no	no	n.d.				
Burkholderia	no	n.d.	yes				
Bacillus	n.d.	n.d.	n.d.				
Lysinibacillus	n.d.	n.d.	n.d.				
Frigoribacterium	n.d.	n.d.	yes				
Pseudomonas	yes	n.d.	n.d.				
Rhizobium	yes	n.d.	n.d.				
Bacillus	n.d.	n.d.	yes				
Paenibacillus	n.d.	n.d.	n.d.				
unknown	no	no	n.d.				

Pseudomonas	n.d.	n.d.	n.d.				
Serratia	no	no	n.d.				
Micrococcus	n.d.	n.d.	n.d.				
Sphingopyxis	n.d.	n.d.	yes				
E. coli	yes	no	n.d.				
Paenibacillus	n.d.	n.d.	n.d.				
Acetobacter	n.d.	n.d.	yes				
Micrococcus	n.d.	no	n.d.				
Micrococcus	n.d.	n.d.	n.d.				
**Apr-2014**				**Oct-2014**			
Pseudomonas	no	no	no	*Staphylococcus*	no	no	0.92
Clavibacter	no	no	no	*Staphylococcus*	no	no	2.00
Arthrobacter	no	no	no	unknown	no	no	0.86
unknown	no	no	15.25	unknown	no	yes	2.75
Micrococcus	no	no	5.25	unknown	no	yes	2.60
Streptomyces	no	no	11.68	unknown	no	no	7.28
Bacillus	no	no	20.96	unknown	no	no	30.00
Micrococcus	no	no	3.14	unknown	no	no	30.00
unknown	no	yes	4.45	*Stenotrophomonas*	no	no	30.00
Bacillus	no	no	3.02	unknown	no	no	no
Bacillus	no	no	no	unknown	no	no	no
Bacillus	no	no	no	unknown	no	no	no
Pseudomonas	no	yes	1.18	*Pseudomonas*	yes	no	1.71
Bacillus	no	no	3.00	unknown	no	no	0.59
Micrococcus	no	no	no	*Pseudomonas*	yes	no	0.48
Micrococcus	no	no	1.25	*Micrococus*	no	no	4.00
Pseudomonas	no	yes	no	unknown	no	no	1.50
Staphylococcus	no	no	1.58	unknown	yes	no	7.00
Bacillus	no	no	0.06	*Pseudomonas*	no	no	9.50
Paenibacillus	no	no	0.04	*Pseudomonas*	no	no	10.00
Pseudomonas	no	no	0.19	*Pseudomonas*	no	no	7.50
Staphylococcus	no	no	173.88	*Pseudomonas*	yes	no	2.30
Kocuria	no	no	17.62	unknown	yes	no	4.80
Bacillus	yes	no	17.54	*Pseudomonas*	yes	no	4.80
Pseudomonas	no	yes	17.66				
**Apr-2015**							
unknown	no	yes	1.00	unknown	no	no	no
unknown	no	yes	1.00	unknown	no	no	no
Pseudomonas	no	no	4.00	unknown	no	no	no
Bacillus	yes	no	3.48	unknown	no	yes	no
Flavobacterium	yes	yes	4.49	unknown	no	no	no
Bacillus	no	no	5.49	*Pseudomonas*	no	no	3.00
unknown	no	yes	2.60	*Micrococcus*	no	yes	18.00
unknown	yes	yes	30.00	*Pseudomonas*	yes	no	12.00
unknown	yes	yes	30.00	*Bacillus*	yes	no	35.00
Pseudomonas	yes	yes	30.00	*Pseudomonas*	yes	no	12.00
unknown	yes	no	no	*Pseudomonas*	yes	no	5.80
unknown	no	no	no	*Pseudomonas*	yes	no	1.20

*n.d., not determined

Table S4: Plant growth promoting abilities of mycorrhiza helper bacteria.

Genus	Siderophore production	Phosphate mobili-zation	IAA (µM/biomass)	Growth on N-free medium
Bacillus cereus MRZ-1	yes	no	3.00 +Trp	yes
Lysinibacillus sp. MRZ-2	no	no	2.45	no
Bacillus pumilus MRZ-3	no	no	2.48	yes
Bacillus zhangzhouensis MRZ-4	no	no	1.56	yes

+ Trp, production only in the presence of additional 0.5 µM tryptophan

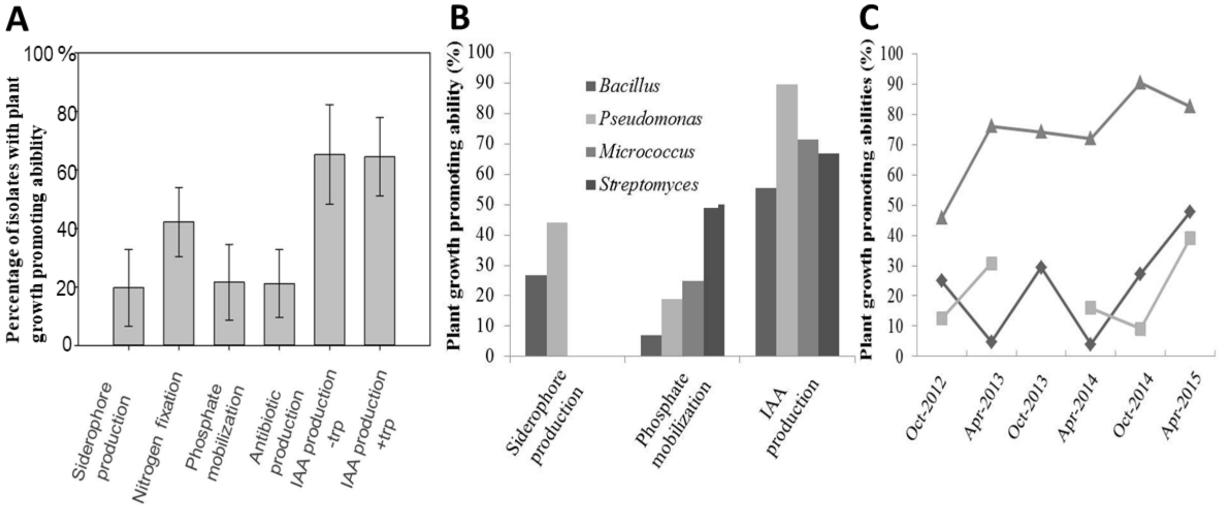

Figure S4: Plant growth promotion features of ectomycorrhizosphere isolates. Isolates of the six sampling times were analyzed for different plant growth promotion features (A), grouped in accordance to their genus (B), and the respective sampling times (C), phosphate mobilization data for October 2013 were not generated. Triangle: IAA production, diamond: siderophore production, square: phosphate mobilization.

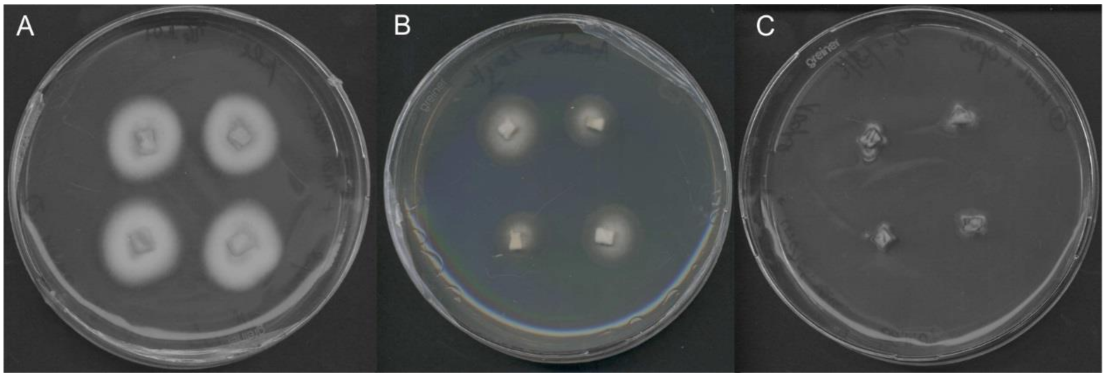

Figure S5: Growth of *T. vaccinum* after 4 weeks on MMNb with 10 g/L glucose (A), on C-deficient MMNb with 40 g/L cellulose and 0.5 g/L glucose, and without malt extract (B), on C-deficient MMNb with 0.5 g/L glucose and without malt extract.

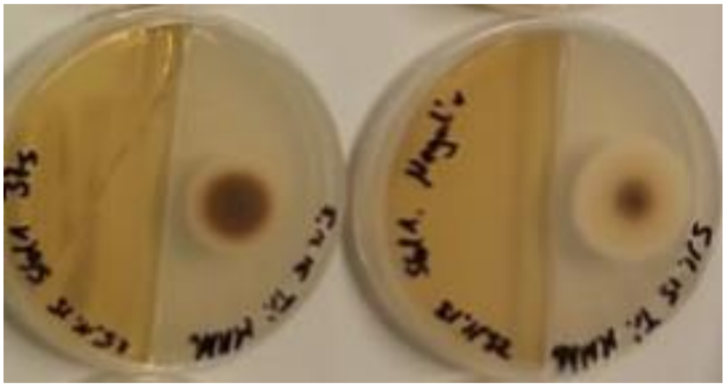

Figure S6: Increased pigmentation of *T. vaccinum* by volatiles of *B. cereus* MRZ-1(left) in comparison to control without volatile treatment (right).

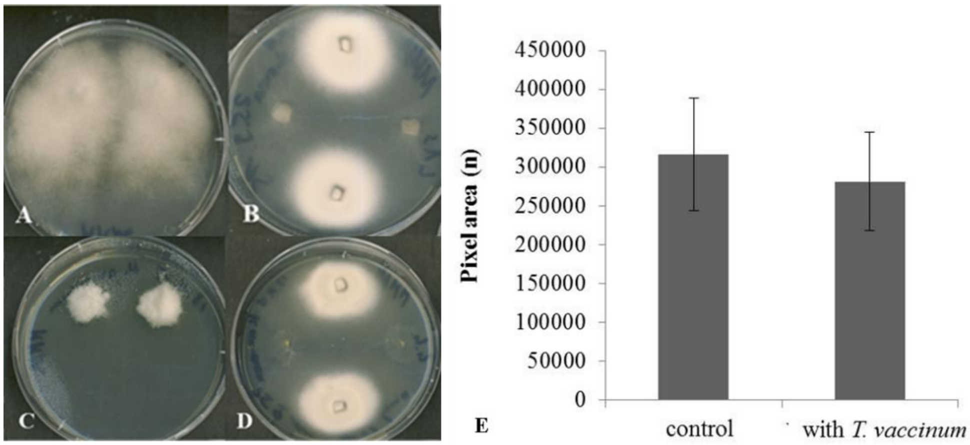

Figure S7: Growth of the pathogens *B. cinerea* (A) and *H. annosum* (C) on MMNb after 10 days; co-cultivation with *T. vaccinum* inoculated on the vertical axis (B, D). Mycelial growth area of *B. cinerea* was analyzed by pixel count (E) with or without *T. vaccinum* grown in divided plates to analyze volatile effects. Error bars indicate standard deviation (n= 3).
